# Food availability, energetic constraints and reproductive development in a wild seasonally breeding songbird

**DOI:** 10.1111/1365-2435.12448

**Published:** 2015-05-01

**Authors:** Scott Davies, Thomas Cros, Damien Richard, Simone L. Meddle, Kazuyoshi Tsutsui, Pierre Deviche

**Affiliations:** ^1^ School of Life SciencesArizona State UniversityTempeArizona85287USA; ^2^ Faculté des Sciences Fondamentales et AppliquéesUniversité de PoitiersPoitiers86022France; ^3^ The Roslin InstituteThe Royal (Dick) School of Veterinary StudiesThe University of EdinburghEaster BushMidlothianEH25 9RGUK; ^4^ Laboratory of Integrative Brain SciencesDepartment of Biology and Center for Medical Life ScienceWaseda UniversityTokyo162‐8480Japan; ^5^ Present address: Department of Biological SciencesVirginia TechBlacksburgVirginia24061USA

**Keywords:** gonad development, gonadotropin‐inhibitory hormone, gonadotropin‐releasing hormone, hypothalamic–pituitary–gonadal axis, luteinizing hormone, neuropeptide Y, reproductive physiology, seasonal reproduction, testosterone

## Abstract

1. In many organisms, food availability is a proximate cue that synchronizes seasonal development of the reproductive system with optimal environmental conditions. Growth of the gonads and secondary sexual characteristics is orchestrated by the hypothalamic–pituitary–gonadal (HPG) axis. However, our understanding of the physiological mechanisms by which food availability modulates activity of the HPG axis is limited.
2. It is thought that many factors, including energetic status, modulate seasonal reproductive activation. We tested the hypothesis that food availability modulates the activity of the HPG axis in a songbird. Specifically, we food‐restricted captive adult male Abert's Towhees *Melozone aberti* for 2 or 4 weeks during photoinduced reproductive development. A third group (control) received *ad libitum* food throughout. We measured multiple aspects of the reproductive system including endocrine activity of all three levels of the HPG axis [i.e. hypothalamic gonadotropin‐releasing hormone‐I (GnRH‐I), plasma luteinizing hormone (LH) and testosterone (T)], and gonad morphology. Furthermore, because gonadotropin‐inhibitory hormone (GnIH) and neuropeptide Y (NPY; a potent orexigenic peptide) potentially integrate information on food availability into seasonal reproductive development, we also measured the brain levels of these peptides.
3. At the hypothalamic level, we detected no effect of food restriction on immunoreactive (ir) GnRH‐I, but the duration of food restriction was inversely related to the size of ir‐GnIH perikarya. Furthermore, the number of ir‐NPY cells was higher in food‐restricted than control birds. Food restriction did not influence photoinduced testicular growth, but decreased plasma LH and T, and width of the cloacal protuberance, an androgen‐sensitive secondary sexual characteristic. Returning birds to *ad libitum* food availability had no effect on plasma LH or T, but caused the cloacal protuberance to rapidly increase in size to that of *ad libitum*‐fed birds.
4. Our results support the tenet that food availability modulates photoinduced reproductive activation. However, they also suggest that this modulation is complex and depends upon the level of the HPG axis considered. At the hypothalamic level, our results are consistent with a role for the GnIH and NPY systems in integrating information on energetic status. There also appears to be a role for endocrine function at the anterior pituitary gland and testicular levels in modulating reproductive development in the light of energetic status and independently of testicular growth.

## Introduction

To maximize reproductive success, organisms often temporally synchronize their breeding period with optimal environmental conditions (Baker 1938; Wingfield & Kenagy 1986; Lindström 1999; Lourdais *et al*. 2002). This synchronization has been demonstrated in a variety of taxa (Munro, Scott & Lam 1990; Boyd 1991; Nager & van Noordwijk 1995; Olsson & Shine 1998; Olive, Lewis & Beardall 2000). In many adult organisms, the reproductive system is regressed outside the breeding period and must recrudesce before the start of the next breeding period. In these organisms, reproductive recrudescence (i.e. regrowth of the gonads and secondary sexual characteristics) is, therefore, an important determinant of the breeding period. This is the case in most birds, which generally exhibit distinct breeding and non‐breeding life‐history stages that are characterized by dramatic changes in reproductive physiology, morphology and behaviour (Williams 2012; Davies & Deviche 2014).

To correctly time reproductive development, organisms use proximate environmental cues to predict favourable conditions (Ims 1990), and one such cue in birds is the annual change in day length (photoperiod). The annual cycle of photoperiod is constant between years at a given location and can predict favourable conditions. Accordingly, birds use photoperiod as the ‘initial predictive cue’ that stimulates the hypothalamic–pituitary–gonadal (HPG) axis to begin reproductive development (Wingfield 1980; Dawson *et al*. 2001) and the mechanism by which increasing vernal photoperiod influences the avian HPG axis has been studied extensively. Light is detected by deep brain photoreceptors, and the circadian system is used to measure the length of this light signal (Follett, King & Meddle 1998; Sharp 2005). Information from these photoreceptors is relayed via the pars tuberalis (PT) of the pituitary gland to increase the production and secretion of the neuropeptide gonadotropin‐releasing hormone‐I (GnRH‐I; King & Millar 1982; Sharp & Ciccone 2005). Gonadotropin‐releasing hormone‐I is the primary secretagogue of the gonadotropins, luteinizing hormone (LH) and follicle‐stimulating hormone (FSH), from the anterior pituitary gland (Kuenzel 2000). These hormones stimulate development of the gonads and secretion of the sex steroids testosterone (T) and oestradiol (E_2_) in males and females, respectively (Murton & Westwood 1977), and these steroids, in turn, stimulate development of secondary sexual organs and promote the expression of reproductive behaviours.

In contrast to photoperiod, many environmental variables often vary from year to year at a given location. Birds may modulate the activity of their reproductive system in the light of supplementary environmental cues, including ambient temperature (Wingfield *et al*. 2003; Schaper *et al*. 2012b), rainfall (Hau *et al*. 2004; Small, Sharp & Deviche 2007), social interactions (Wingfield & Wada 1989; Maney, Goode & Ball 2007; Small *et al*. 2008a; Stevenson *et al*. 2008) and food availability (Lack 1968; Hau, Wikelski & Wingfield 2000; Hahn *et al*. 2005; O'Brien & Hau 2005). Although considerable information is available about the effects of day length on the HPG axis, much less is known about the physiological mechanisms by which these non‐photic environmental factors influence reproductive activity and most studies to date have focused primarily on the pituitary gland. Food availability, in particular, is thought to modulate reproductive activity partly through an individual's energetic status. Accordingly, within the window of opportunity for breeding determined by day length, a bird's energetic status is predicted to constrain the timing of reproductive development (Drent & Daan 1980; Wingfield & Kenagy 1986; Meijer & Drent 1999; Hahn *et al*. 2005). Thus, birds in good energetic status can begin this development shortly after stimulation by sufficiently long days, whereas birds in poor energetic status delay development until they have acquired sufficient energy stores. Various experimental and correlative approaches, including food supplementation (Schoech 1996, 2009; Scheuerlein & Gwinner 2002; Harrison *et al*. 2010) and natural variation in the abundance of wild food sources (Ligon 1974; Hahn 1998), found that increased food availability is associated with earlier seasonal breeding in wild birds (reviewed by Davies & Deviche 2014). Captive studies on the effect of food restriction or deprivation on the avian reproductive system have, however, yielded inconsistent results (Dawson 1986; Meijer 1991; Hahn 1995; Perfito *et al*. 2008).

A candidate neuropeptide that may integrate information on food availability and fine‐tune seasonal reproductive development is gonadotropin‐inhibitory hormone (GnIH; Tsutsui *et al*. 2000) and its links with hypothalamic cells that produce neuropeptide Y (NPY; McConn *et al*. 2014). Across vertebrates, GnIH opposes the effect of GnRH on the HPG axis (Greives *et al*. 2008) and inhibits reproductive function by acting on hypothalamic GnRH neurons, anterior pituitary gland gonadotropes and gonads (Tsutsui *et al*. 2010, 2012, 2013; Clarke 2011; Clarke & Parkington 2013; Davies & Deviche 2014). In addition, GnIH has an orexigenic effect in birds and mammals (Tachibana *et al*. 2005, 2008; Clarke *et al*. 2012; Clarke & Parkington 2013; Tsutsui *et al*. 2013; McConn *et al*. 2014). In mammals, GnIH neurons project to hypothalamic regions that regulate appetite and energetic status (Qi, Oldfield & Clarke 2009; Ubuka *et al*. 2009). These regions contain NPY‐producing neurons, and the orexigenic action of GnIH may, therefore, be mediated by these neurons (Clarke *et al*. 2009). Neuropeptide Y is among the most potent endogenous orexigenic factors (Boswell 2001; Hill, Elmquist & Elias 2008; Pralong 2010), and, in mammals, NPY‐producing cells integrate information on energetic status via both hormones and metabolites (Marty, Dallaporta & Thorens 2007). Indeed, reciprocal projections from NPY cells to GnIH cells and evidence from mammalian studies suggest that GnIH cells modulate their activity in response to energetic status (Klingerman *et al*. 2011). Therefore, the GnIH–NPY system appears to simultaneously regulate the activity of the HPG axis and food intake in response to energetic status and may play a role in the modulation of reproductive development by food availability (Davies & Deviche 2014).

Here, we investigated whether food availability modulates reproductive development in a seasonally breeding songbird and examined potential neuroendocrine mechanisms mediating this modulation. Specifically, we hypothesized that energetic status influences reproductive development. To test this hypothesis, we compared hypothalamic levels of GnRH, plasma LH and T, testicular development and cloacal protuberance width of adult male Abert's Towhees *Melozone aberti* (Baird) subjected to various food availability regimes during photoinduced gonadal development. We predicted that birds with access to *ad libitum* food and, hence, in good energetic status would undergo reproductive activation at a faster rate than food‐restricted birds in poor energetic status. We also aimed to shed light on the physiological mechanism(s) by which food availability modulates the HPG axis and, for this, quantified hypothalamic levels of GnIH and NPY in response to food availability. To our knowledge, this study is the first in a wild bird species to examine the effects of food restriction on the endocrine regulation of reproductive development at all levels of the HPG axis simultaneously. A better understanding of the physiological mechanisms by which food availability modulates seasonal reproductive activation will improve our understanding of how variation in this environmental cue is transduced into annual variation in the timing of breeding periods.

## Materials and methods

### Bird Capture and Housing

During January 2011, we caught 24 adult male Abert's Towhees from Robbins Butte Wildlife Area, Maricopa Co., AZ (altitude: 249 m; latitude: 33°19′N; longitude: 112°38′W), using mist nets and conspecific song playback. We determined sex and age using behaviour (singing and aggressive response to conspecific playback in males only), skull pneumatization and wing chord (wing chord ≥92 mm = male; Pyle 1997). We transported birds to Arizona State University and randomly divided them between two environmental chambers, both maintained at 23 °C (±1 °C) with a short light cycle (9 L : 15 D; lights on at 06:00). We individually housed birds in 76 × 46 × 46 cm cages with opaque barriers on three sides of each cage and provided *ad libitum* food (Mazuri small bird maintenance diet) and water. To permit individual identification, we marked each bird with a numbered aluminium tarsal band. After a 1‐week acclimation period in captivity, we increased the photoperiod to 14 L (lights on at 06:00) to induce reproductive development. To measure the average daily *ad libitum* food consumption of each individual over 2 weeks, we provided a pre‐weighed amount of food shortly after the lights went on and weighed the remaining food (including spillage on the cage bottom) 24 h later. To minimize spillage, we partially covered food dishes with tape, allowing only a small opening to access food. All procedures were approved by the Arizona State University Institutional Animal Care and Use Committee and conducted under appropriate scientific collecting permits issued by the Arizona Game and Fish Department and the US Fish and Wildlife Service.

### Experimental Protocol

**Figure 1:**
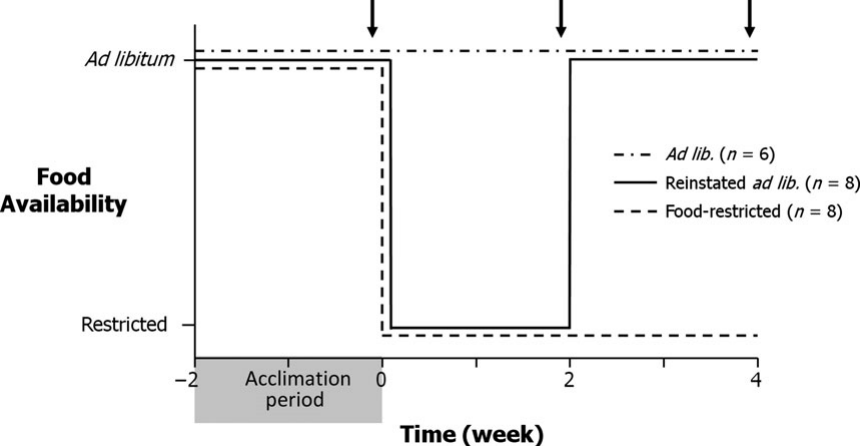
Schematic representation of the food availability regime. During the acclimation period, we quantified the daily food consumption of each bird. Food‐restricted birds received 70% of their individual *ad libitum* consumption. Photoperiod for the duration of the experiment shown in the schematic was 14 L : 10 D throughout. Arrows indicate the collection of blood samples and morphometric data. Following the third collection period, we also collected the brain and testes from each bird.

Following the 2‐week *ad libitum* food consumption period, we randomly assigned birds to one of three groups (*n *=* *8 birds per group): (i) *ad libitum* food, (ii) restricted food availability for 4 weeks (see more below) or (iii) 2 weeks of food restriction followed by 2 weeks of *ad libitum* food (Fig. 1). We used a stratified random assignment of treatment groups to spatially intersperse the treatments across the two environmental chambers, such that each environmental chamber contained an equal number of birds from each treatment group (i.e. 12 birds per room, with four birds per group). Two birds in the *ad libitum* food group died during the study due to unknown causes, reducing this group size to six.

To ensure that the food restriction regime caused a marked and consistent reduction in body mass, we food‐restricted birds with the goal of reducing their body mass to 85% of their *ad libitum* mass. This target body mass is comparable to that used by Lal *et al*. (1990) in similar studies on cockerels (*c. *83% of *ad libitum* mass) and on European Starlings *Sturnus vulgaris* (*c. *80% of *ad libitum* mass; Meijer 1991). To reach this target weight and based on a separate pilot study on six Abert's Towhees, experimental birds received 70% of their individual average *ad libitum* food consumption. We weighed all birds daily immediately prior to dispensing the daily food ration. If a bird's mass decreased below its 85% target mass, this bird was immediately fed the difference between its current mass and its target mass (Morrison *et al*. 2002). By using this individually customized, flexible restriction regime, we could control food availability and concurrently confirm that the body mass of each bird was reduced and stabilized at the target reduction.

From each bird, we collected a blood sample to quantify plasma LH and T immediately prior to (i) the switch to food restriction (defined as week 0), (ii) the switch back to *ad libitum* food (week 2) and (iii) the end of the study (week 4; Fig 1). We collected blood (*c. *200 μL) from the right jugular vein using a heparinized syringe within 2 min of removing a bird from its cage. The sample was then placed on ice and centrifuged within 1 h. We harvested plasma using a Hamilton glass syringe and froze aliquots at −80 °C until assayed. Immediately following collection of each blood sample, we quantified the furcular fat stores and size of the pectoral muscles of all the birds to estimate their energetic condition. We visually estimated the amount of furcular fat by assigning a score of 0–5 (a score of 0 representing no fat, 5 representing bulging fat deposits; Helms & Drury 1960). Furthermore, because the pectoral muscles in birds are the largest store of protein and muscle protein can be converted into energy via gluconeogenesis, we also estimated the size of the pectoral muscles on a scale ranging from 0 to 3 (adapted from Gosler 1991; 0 representing concave pectoral muscles and a prominent keel, 3 representing convex pectoral muscles that protruded above the keel; Salvante, Walzem & Williams 2007). At this time, we also measured the width of the cloacal protuberance (±0·1 mm; using digital calipers), an androgen‐sensitive secondary sexual characteristic. We staggered the collection of blood samples and morphometrics over two consecutive days by randomly assigning towhees to one of four groups, with six birds in each group. We collected blood samples for any given group always in the same order beginning at 09:00 AM and noted the time that each blood sample was taken.

### Perfusion and Tissue Collection

To investigate the effect of food availability on the central control of reproduction and testicular development, at the end of the study, we collected the brain and testes of each bird. Following deep anaesthesia induced by intramuscular injection (250 μL into each pectoral muscle) of a ketamine/xylazine cocktail [ketamine: 8 mg per 0·5 mL (160 mg kg^−1^); xylazine: 160 μg per 0·5 mL (3·2 mg kg^−1^) dissolved in 0·9% NaCl solution], we transcardially perfused birds with 35 mL of wash solution (0·9% NaCl and 0·1% NaNO_2_ in 0·1 m phosphate buffer, PB), followed by 35 mL of fixative (4% paraformaldehyde and 0·1% NaNO_2_ in 0·1 m PB). We then decapitated birds, exposed the brain and placed heads in the same fixative overnight at 4 °C. We also collected both testes and removed all extra connective tissue before weighing to the nearest 0·1 mg and post‐fixed the tissue as described above for brains. The day after perfusion, we dissected brains out of the skull and post‐fixed them overnight (4% paraformaldehyde and 0·1% NaNO_2_ in 0·1 m PB). Brains and testes were gelatin‐embedded and cryoprotected according to a modification of a previously published protocol (Saldanha, Deviche & Silver 1994; Small *et al*. 2008b) and stored at −80 °C until sectioned.

We coronally sectioned brains at a thickness of 30 μm using a cryostat at −21 °C, with the stereotaxic atlas of the canary *Serinus canaria* brain as a reference (Stokes, Leonard & Nottebohm 1974). Sections were divided into four parallel series by systematically alternating between four separate six‐well Falcon plates containing cryoprotectant solution (Watson *et al*. 1986) and stored in cryoprotectant at −20 °C until immunolabelled. One series was used for each assay (cGnRH‐I, GnIH or NPY).

### GnRH, GnIH and NPY Immunocytochemistry

**Figure 2:**
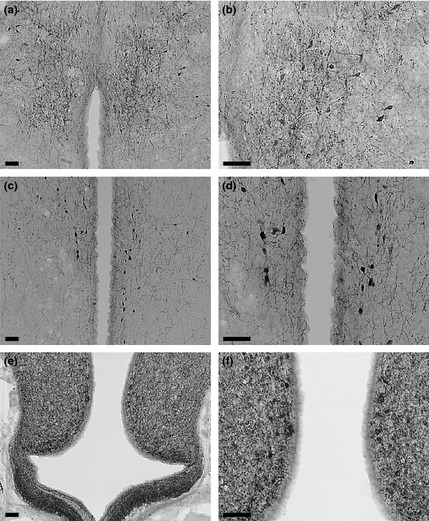
Representative photomicrographs of immunolabelled coronal brain sections of male Abert's Towhees *Melozone aberti* under lower magnification (left column) and higher magnification (right column). (a and b) immunoreactive gonadotropin‐releasing hormone‐I (GnRH‐I) perikarya in the pre‐optic area. (c and d) immunoreactive gonadotropin‐inhibitory hormone (GnIH) perikarya in the paraventricular nucleus. (e and f) immunoreactive neuropeptide Y (NPY) perikarya in the infundibular nucleus. Scale bars = 50 μm.

We immunostained brain sections for GnRH, GnIH and NPY in two assays per peptide, with sections from randomly selected birds in each assay. Previous studies have determined the distribution of these peptides in the avian brain. Gonadotropin‐inhibitory hormone‐I is primarily synthesized in the pre‐optic area (POA; Parry *et al*. 1997; Dawson & Goldsmith 1997), and GnIH is synthesized solely in the paraventricular nucleus (PVN; Tsutsui *et al*. 2000; Osugi *et al*. 2004; Tsutsui *et al*. 2010). Neuropeptide Y‐producing neurons are widely distributed throughout the avian brain, but the only cell population that responds to energetic status is located in the infundibular nucleus (IN; Kuenzel & McMurtry 1988; Boswell 2001; Boswell, Li & Takeuchi 2002). Therefore, from each bird, we randomly selected sections covering the entire POA, PVN and IN for GnRH, GnIH and NPY, respectively; Fig. 2). For GnIH and NPY, we stained an average of 10 sections per bird and for GnRH, an average of six sections per bird. Since sections were randomly selected from each bird, we assumed that this sampling design gives an unbiased estimate of the number of immunoreactive cells for a given bird and, therefore, calculated the median number of cells per section for each bird.

The GnRH and GnIH staining protocols have been previously published and validated in our laboratory (Small *et al*. 2008b). The specificity of the NPY antiserum has been established in the chicken (Kuenzel & McMurtry 1988) and Ring Dove *Streptopelia risoria* (Strader & Buntin 2001). Pre‐absorption of the NPY antiserum with human/rat NPY (H‐6375; Bachem, Torrance, CA, USA) before application to Abert's Towhee brain sections abolished the staining. Following two washes in 0·1 m PB for 30 min, we serially exposed sections to 0·36% H_2_O_2_, washed them 3 × 5 min in 0·1 m PB, blocked background immunoreactivity for 1 h and incubated sections overnight in primary antiserum. We then washed sections three times for 10 min in PB with 0·1% Triton X‐100 (Sigma‐Aldrich Co., St. Louis, MO, USA; 0·1% PBT), incubated for 1 h in secondary antibody, washed three times for 10 min in 0·1% PBT, incubated for 1 h in avidin–biotin complex (ABC Vectastain Elite kit; Vector Laboratories, Burlingame, CA, USA), washed three times for 15 min in 0·1% PBT, incubated in Vector SG peroxidase chromagen for 2 min and washed twice for 5 min in 0·1 m PB. After mounting on glass microscope slides, we allowed immunolabelled sections to dry at room temperature for 24 h before dehydrating through a graded ethanol series, clearing in xylene and coverslipping using Permount mounting medium (Fisher Scientific, Pittsburg, PA, USA).

#### GnRH

We used anti‐cGnRH‐I rabbit polyclonal antiserum (6DL31/4 prepared by P.J. Sharp) at a dilution of 1 : 10 000 in 0·3% PBT. To block non‐specific sites, we used normal rabbit serum (Vector Laboratories, Inc.; 1 : 200 in 0·3% PBT), and we used biotinylated rabbit anti‐sheep IgG (Vector Laboratories, Inc.; 1 : 200 in 0·3% PBT) as a secondary antibody.

#### GnIH

We used anti‐Japanese Quail GnIH antiserum (Tsutsui *et al*. 2000) at a dilution of 1 : 5000 in 0·3% PBT. We used normal horse serum (Vector Laboratories; 1 : 30 in 0·3% PBT) to block non‐specific sites and biotinylated mouse/rabbit IgG (Vector Laboratories; 1 : 100 in 0·3% PBT) as a secondary antibody.

#### NPY

We used anti‐human/rat NPY antiserum (T‐4070; Bachem) at a dilution of 1 : 10 000 in 0·3% PBT. We used normal goat serum (Vector Laboratories; 1 : 30 in 0·3% PBT) to block non‐specific sites and biotinylated rabbit IgG (Vector Laboratories; 1 : 100 in 0·3% PBT) as a secondary antibody.

### Immunocytochemistry Data Collection

All data were collected without knowledge of the treatment group. For each bird, we counted the number of cells immunoreactive for GnRH‐I, GnIH and NPY present in each immunostained section. We quantified the area and optical density of GnRH and GnIH immunolabelled perikarya using digital photographs taken at 400× magnification with an Olympus DEI‐750D digital camera mounted on an Olympus BX60 light microscope (Olympus Optical Co., Ltd., Tokyo, Japan). Due to the dense network of NPY‐ir fibres in the IN (Fig. 2), perikaryon area and optical density could not be accurately quantified for this peptide. Light intensity, aperture diameter and camera shutter speed were standardized for all image captures. We photographed five randomly selected perikarya from each section. Only perikarya for which the entire perimeter was unobstructed and clearly visible were used; perikarya with overlapping structures, such as other perikarya, were not analysed. Digitized images were analysed using Image‐Pro Plus (Media Cybernetics, LP, Silver Spring, MD, USA) by manually outlining each perikaryon and then determining the immunolabelled area and optical density (arbitrary units: 0 = no staining, complete light transmission; and 1 = complete staining saturation, no light transmission) of each. All images were standardized for individual variations in background immunolabelling using Image‐Pro's background correction function.

To determine the density of GnRH‐I‐ir and GnIH‐ir fibres in the median eminence (ME), we took pictures from two sections per brain. We corrected for background staining of each image as described above. On the resulting image, we used Image‐Pro Plus to measure the optical density of five areas of interest (AOI, 65 × 65 μm each) per brain section. Areas of interest were evenly spaced from left to right along the ventral edge of the ME. We calculated an average optical density for each section, then an average for each bird.

### Testicular Morphology

We sectioned testes at a thickness of 30 μm using a cryostat at −21 °C and stored sections in 0·1 m PB until mounting on glass microscope slides later the same day. After allowing sections to dry at room temperature for 24 h, we rehydrated them through a graded ethanol series before staining with haematoxylin (S212A; Poly Scientific, Bay Shore, NY, USA) for 3 min. We then rinsed the sections for 5 min under running tap water before destaining by dipping them in acid ethanol ten times. Following another 2 min rinse with tap water, we stained sections with eosin (S176, Poly Scientific) for 30 s, dehydrated them through a graded ethanol series, cleared them in xylene and coverslipped using Permount.

Vernal reproductive development in many seasonally breeding birds involves a marked increase in testis size caused by increases in the length and diameter of seminiferous tubules. Seminiferous tubule diameter is, therefore, a sensitive indicator of testicular exocrine function (Amann 1986; Jenkins, Ross & Young 2007). We randomly selected eight sections from each bird (four from each testis) and, using Image‐Pro Plus, measured the shortest diameter of 10 seminiferous tubules per section randomly selected using a grid overlaid on the image. These measurements were used to calculate an average seminiferous tubule diameter for each bird. We also recorded when spermatozoa were present in the seminiferous tubules.

### Hormone Assays

To measure plasma LH, we used the radioimmunoassay described previously (Sharp, Dunn & Talbot 1987), with slight modifications. Briefly, the assay reaction volume was 60 μL, comprised of 20 μL of plasma sample or standard, 20 μL of primary rabbit LH antibody and 20 μL of I^125^‐labelled LH. The primary antibody was precipitated to separate free and bound I^125^ label using 20 μL of donkey anti‐rabbit precipitating serum and 20 μL of non‐immune rabbit serum. All samples were measured in duplicate in a single assay. The intra‐assay coefficient of variation was 3·6% and the minimum detectable dose was 0·2 ng mL^−1^. This radioimmunoassay has been used extensively to quantify plasma LH in many avian species (Lal *et al*. 1990; Lea, Talbot & Sharp 1991; Malecki *et al*. 1998; Ciccone, Dunn & Sharp 2007; Schaper *et al*. 2012a; Fraley *et al*. 2013), including multiple Emberizidae sparrows (Meddle *et al*. 2002; Deviche, Sabo & Sharp 2008; Deviche *et al*. 2012a,b; Wingfield *et al*. 2012).

To quantify plasma T, we used competitive enzyme‐linked immunoassay kits, according to the manufacturer's instructions after 8× dilution in assay buffer containing steroid displacement reagent (Enzo Life Sciences, Ann Arbor, MI, USA). This assay has been validated for Abert's Towhee in our laboratory (Fokidis, Orchinik & Deviche 2009). We assayed samples in duplicate and randomly assigned them to assay plates, except that all samples collected from any given individual were assayed on the same plate. Each plate included a complete standard curve. The average assay sensitivity was 9·4 pg mL^−1^. The average intra‐ and interassay coefficients of variation were 6·9% and 3·7%, respectively (*n *=* *2 plates; 66 samples). The primary antibody used in this assay has <5% cross‐reactivity with 17β‐oestradiol, 5α‐dihydrotestosterone (DHT), corticosterone and progesterone (manufacturer's specifications).

### Statistical Analyses

To analyse the effects of food availability (*ad libitum*, reinstated *ad libitum* or restricted) on body mass, furcular fat score, pectoral muscle score, cloacal protuberance width, and plasma LH and T, we used a two‐way analysis of variance with repeated measures (rmanova). The sphericity assumption was tested using Mauchly's test, and if this was violated, we used the Greenhouse‐Geisser correction. To analyse the effect of food availability on the number of GnRH, GnIH and NPY cells, we used Kruskal–Wallis test followed by Dunn's pairwise comparison. Gonadotropin‐releasing hormone‐I and GnIH measures (median number of ir cells, cell body area and optical density, and optical density of fibres in the ME), and seminiferous tubule diameter were compared using one‐way anova. We performed all statistical analyses using pasw version 18.0 (Chicago, Illinois, USA) with an alpha of 0·05 on untransformed data, with the exception of cloacal protuberance width data that were log‐transformed to attain normality. *Post hoc* comparisons for anovas were performed using Tukey's honestly significant difference (HSD) test. Data analysed using parametric methods are presented as means ± standard error of the mean (SEM), and data analysed using nonparametric methods are presented as medians ± interquartile range (IQR). All graphs depict untransformed data.

## Results

### Body Mass

**Figure 3:**
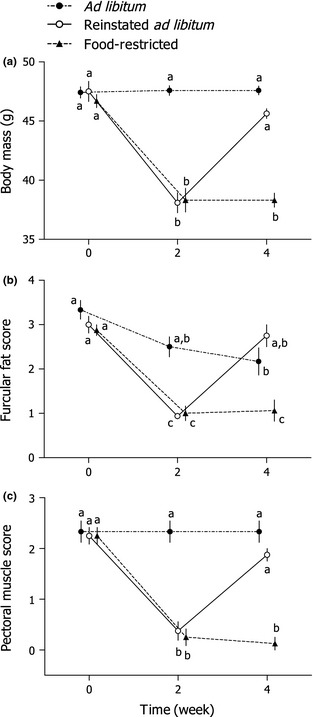
Body mass, furcular fat and pectoral muscles were modulated by food availability in adult male Abert's Towhees *Melozone aberti*. Body mass (a), furcular fat score (b) and pectoral muscle score (c) were reduced following 2 weeks of food restriction (i.e. 70% of *ad libitum* consumption), and returning birds to *ad libitum* availability increased these parameters to levels similar to those of the control group. For details of study design and sample sizes, see Fig. 1 and the Materials and methods section. Data points are means ± SEM, and points with identical letters are not significantly different (*P *>* *0·05; Tukey honestly significant difference test). For visual clarity, points have been separated along the horizontal axis.

Body mass was significantly affected by food availability (*F*_2,19_ = 32·68, *P *<* *0·0001), time (*F*_2,38_ = 93·34, *P *<* *0·0001) and the interaction between these factors (*F*_4,38_ = 39·12, *P *<* *0·0001; Fig. 3). Body mass of *ad libitum*‐fed birds was similar throughout the experiment (Tukey HSD, *P *≥* *0·05). Two weeks of food restriction caused body mass to decrease in both the reinstated *ad libitum* and the food‐restricted groups (Tukey HSD, *P *≤* *0·05). Body mass did not decrease further in food‐restricted birds, but returning birds to *ad libitum* food availability for 2 weeks resulted in a body mass increase to levels similar to those at the beginning of the study (Tukey HSD, *P *<* *0·05).

### Fat Score

Furcular fat scores were significantly affected by food availability (*F*_2,19_ = 10·78, *P *=* *0·001), time (*F*_2,38_ = 74·56, *P *<* *0·0001) and the interaction between these factors (*F*_4,38_ = 15·25, *P *<* *0·0001; Fig. 3). Fat scores of birds exposed to *ad libitum* food availability decreased over the 4‐week study (Tukey HSD, *P *<* *0·05). Two weeks of food restriction caused fat scores to further decrease in both the reinstated *ad libitum* and the food‐restricted groups (Tukey HSD, *P *<* *0·05). Fat scores did not decrease further in birds maintained on restricted food availability for another 2 weeks, but returning birds to *ad libitum* food availability for 2 weeks caused fat score to increase to levels similar to those at the beginning of the study (Tukey HSD, *P *<* *0·05).

### Pectoral Muscle Score

Pectoral muscle scores were significantly affected by food availability (*F*_2,19_ = 10·94, *P *<* *0·001), time (*F*_2,38_ = 57·08, *P *<* *0·001) and the interaction between these factors (*F*_4,38_ = 24·72, *P *<* *0·001; Fig. 3). Pectoral muscle scores of birds exposed to *ad libitum* food availability were similar throughout the experiment (Tukey HSD, *P *<* *0·05). Two weeks of food restriction caused pectoral muscle score to decrease in both the reinstated *ad libitum* and the food‐restricted groups (Tukey HSD, *P *<* *0·05). Pectoral muscle score did not decrease further in birds maintained on restricted food availability, but returning birds to *ad libitum* food availability for 2 weeks caused it to increase to levels similar to those at the beginning of the study (Tukey HSD, *P *<* *0·05).

### GnRH

**Figure 4:**
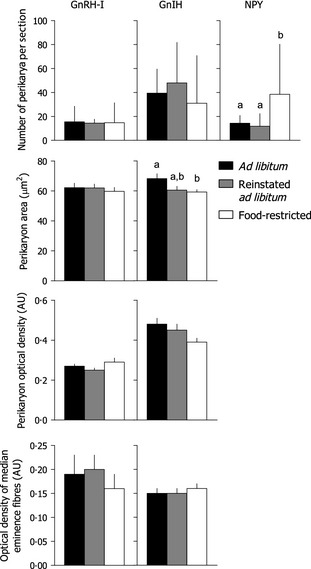
The number, area and optical density of perikarya and optical density of fibres in the median eminence (ME) immunolabelled for gonadotropin‐releasing hormone‐I (GnRH‐I), gonadotropin‐inhibitory hormone (GnIH) and neuropeptide Y (NPY) of adult male Abert's Towhees *Melozone aberti* following food availability treatment. Birds were exposed to *ad libitum* food availability for 4 weeks (*n* = 6; ‘*ad libitum*’), 2 weeks of food restriction (70% of *ad libitum* consumption) followed by 2 weeks of *ad libitum* food (*n* = 8; ‘reinstated *ad libitum*’) or restricted food availability for 4 weeks (*n* = 8; ‘food‐restricted’). Superscript letters indicate significant differences between the groups (*P *<* *0·05; Tukey honestly significant difference test). AU, arbitrary units. Data presented are means (±SEM), with the exception of the number of cells, which are presented as medians and interquartile range.

We found no effect of food availability treatment on GnRH cells, including the number of GnRH‐I‐ir perikarya per section (*H* = 0·54, 2 d.f.*, P* = 0·76), the perikaryon area (*F*_2,21_ = 0·24, *P* = 0·79) or optical density (*F*_2,21_ = 2·07, *P* = 0·15), or the optical density of ME GnRH‐I‐ir fibres (*F*_2,21_ = 0·40, *P* = 0·68; Fig. 4).

### GnIH

GnIH‐ir perikaryon area was influenced by the experimental treatments (*F*_2,21_ = 3·67, *P* = 0·045). *Ad libitum* birds had greater GnIH‐ir perikaryon area than food‐restricted birds (Tukey HSD, *P *<* *0·05). However, there was no effect of food availability on the number of GnIH‐ir cells (*H* = 0·87, 2 d.f., *P* = 0·65), the optical density of GnIH‐ir perikarya (*F*_2,21_ = 3·37, *P* = 0·056) or the density of ME GnIH‐ir fibres (*F*_2,21_ = 0·70, *P* = 0·51; Fig. 4).

### NPY

The number of NPY‐ir cells was a function of food availability treatment (*H* = 7·56, 2 d.f., *P* = 0·023; Fig. 4), with food‐restricted birds having more NPY‐ir cells than *ad libitum* or reinstated *ad libitum* birds (Dunn's method, *P *<* *0·05).

### Plasma Luteinizing Hormone

**Figure 5:**
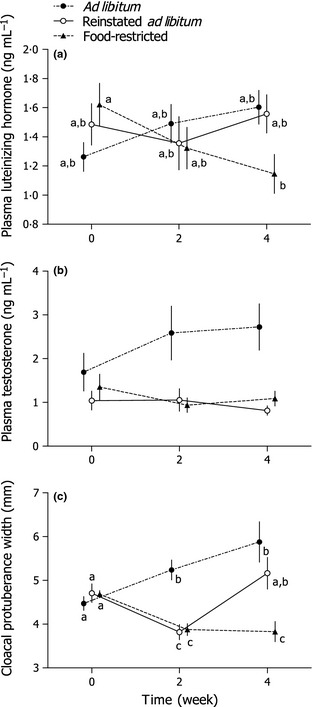
Plasma luteinizing hormone, plasma testosterone and cloacal protuberance width were modulated by food availability in adult male Abert's Towhees *Melozone aberti*. Plasma luteinizing hormone (a) decreased between weeks 0 and 4 in food‐restricted birds. Plasma testosterone (b) was lower in birds exposed to two (reinstated *ad libitum*) or four (restricted) weeks of food restriction (70% of *ad libitum* consumption) compared to control birds with *ad libitum* food availability. Cloacal protuberance width (c) was reduced by food restriction and returning birds to *ad libitum* food availability increased it to a size similar to those of the control group. Points with identical letters are not significantly different (*P *>* *0·05; Tukey honestly significant difference test). For details of study design, see Fig. 1 and the Materials and methods section. Data points are means ± SEM. For visual clarity, points have been separated along the horizontal axis.

There was no overall effect of food availability treatment on plasma LH (*F*_2,19_ = 0·29, *P* = 0·75), nor was there an overall effect of time (*F*_1·5,28·6_ = 0·25, *P* = 0·72). However, there was an interaction between treatment and time (*F*_3,28·6_ = 3·39, *P* = 0·031; Fig. 5). *Post hoc* tests revealed that plasma LH did not change over time in either the *ad libitum* or the reinstated *ad libitum* birds (Tukey HSD, *P'*s > 0·05), but plasma LH of food‐restricted birds decreased between weeks 0 and 4 (Tukey HSD, *P *<* *0·05).

### Testicular Physiology and Morphology

There was an effect of food availability treatment on plasma T (*F*_2,19_ = 10·97, *P* = 0·001), but there was no effect of time (*F*_2,38_ = 0·042, *P* = 0·96) or an interaction between these factors (*F*_4,38_ = 1·33, *P* = 0·28; Fig. 5). *Post hoc* tests revealed that plasma T of the *ad libitum*‐fed birds was higher than both reinstated *ad libitum* and food‐restricted groups (Tukey HSD, *P *<* *0·05), and the latter two groups had similar plasma T (Tukey HSD, *P *> 0·05). Plasma T in the reinstated *ad libitum* and food‐restricted groups did not differ at any point (Tukey HSD, *P *>* *0·05). At the time of sacrifice, there was no effect of treatment on paired testis weight (*F*_2,21_ = 2·21, *P* = 0·14; Table 1) or seminiferous tubule diameter (*F*_2,21_ = 0·61, *P* = 0·56; Table 1). Furthermore, spermatozoa were present in the seminiferous tubules of all birds.

**Table 1:** Photoinduced testicular development of adult male Abert's Towhees *Melozone aberti* was not affected by food availability. Towhees were exposed to either *ad libitum* food availability for 4 weeks (‘*ad libitum*’), 2 weeks of food restriction (70% of *ad libitum* consumption) followed by 2 weeks of *ad libitum* food (‘reinstated *ad libitum*’) or restricted food availability for 4 weeks (‘food‐restricted’) before we collected testes. Data presented are means (± SEM)

	Food availability treatment group			Statistics
	*Ad libitum*	Reinstated *ad libitum*	Food‐restricted	*P*‐value
Paired testis mass (mg)	348·8 (±18·1)	357·0 (±30·1)	294·7 (±17·0)	0·14
Seminiferous tubule diameter (μm)	489·1 (±17·1)	479·1 (±14·2)	462·9 (±18·4)	0·56

### Cloacal Protuberance

Cloacal protuberance width was significantly affected by food availability (*F*_2,19_ = 7·96, *P* = 0·003), time (*F*_2,38_ = 8·13, *P* = 0·001) and the interaction between these factors (*F*_4,38_ = 13·68, *P *<* *0·001; Fig. 5). Cloacal protuberance width of birds exposed to *ad libitum* food availability increased during the first 2 weeks of the experiment (Tukey HSD, *P *<* *0·05), but did not increase further during the last 2 weeks. Two weeks of food restriction caused cloacal protuberance width to decrease in both the reinstated *ad libitum* and the food‐restricted groups (Tukey HSD, *P *<* *0·05). Cloacal protuberance width did not decrease further in birds maintained on restricted food availability, but returning birds to *ad libitum* food availability for 2 weeks caused cloacal protuberance width to increase to levels similar to those of the *ad libitum* birds.

## Discussion

It has long been recognized that food availability plays a crucial proximate role in the development of gonads and secondary sexual characteristics of seasonally breeding birds (Lack 1968; Hahn *et al*. 2005; Williams 2012), but the mechanism mediating this role remains unclear. Food availability is thought to modulate reproductive development partly via energetic status. Limited food availability may constrain this development because birds lack the necessary energy stores for tissue growth and hormone production (Drent & Daan 1980; Wingfield & Kenagy 1986; Meijer & Drent 1999; Hahn *et al*. 2005). Studies in controlled laboratory conditions aimed at testing whether food availability modulates reproductive development through its effects on energetic status must, therefore, ensure that food availability treatments produce a disparity in the energetic condition. To that end, we measured body mass and energy stores (as estimated by furcular fat stores and pectoral muscle size). All of these parameters were, indeed, reduced by the food restriction regime, indicating that food‐restricted birds were in lower energetic condition than control birds. Furthermore, returning birds to *ad libitum* food availability caused all of these parameters to increase to levels similar to those at the beginning of the study. Therefore, the food availability treatments resulted in three groups of birds that differed in the duration that they experienced reduced energetic status.

### Testicular Growth and Endocrine Activity

Despite differences in energetic status, Abert's Towhees in the three experimental groups had similar testis masses and seminiferous tubule diameters. Furthermore, the seminiferous tubules of all towhees contained spermatozoa. If testis growth and spermatogenesis are energetically expensive processes, we would have expected the energetic constraint imposed by food restriction to reduce photoinduced testis growth. As we detected no such reduction, our observations are consistent with the proposition that testis growth in the Abert's Towhee may not be particularly energetically demanding. This conclusion is consistent with results of a study by Caro & Visser (2009) in which Great Tits *Parus major* exposed to ambient temperatures of 8 °C or 22 °C during photoperiodically induced reproductive development had different basal metabolic rates, but showed similar testicular growth. We should point out that neither our study nor that of Caro & Visser (2009) can exclude the possibility that testis growth is energetically demanding, but when energy is limited, birds continue to allocate energy to this process at the cost of other processes. The energetic costs of testis growth and maintenance are notoriously difficult to quantify (Vézina & Salvante 2010). Estimates of the metabolic cost of testis growth based solely on tissue energy content are consistent with the proposition that this process is energetically inexpensive (Walsberg 1983). However, as others have pointed out (Vézina & Salvante 2010), these estimates fail to account for the costs of tissue synthesis, maintenance and function. Indeed, the potential costs of testicular function, particularly increased T production and secretion, are likely to be high.

Testosterone plays a key role in regulating life‐history trade‐offs in male vertebrates and promotes investment in sexual traits, which generally comes at a cost to somatic maintenance. Elevated plasma T during breeding promotes expression of male reproductive behaviours, such as singing, courtship, territorial aggression and mate guarding (Foerster *et al*. 2002; Hau 2007; Kurvers *et al*. 2008). These behaviours by themselves confer energetic costs (Oberweger & Goller 2001; Ward, Speakman & Slater 2003; Ward & Slater 2005; Hasselquist & Bensch 2008), which are likely compounded by indirect costs through reduction in the time spent at rest and for self‐maintenance behaviours (Lynn *et al*. 2000) and foraging (Thomas *et al*. 2003). Behaviours that are stimulated by T also increase predation risk (Schmidt & Belinsky 2013) and decrease survival (Reed *et al*. 2006). Furthermore, investment in T‐dependent sexual traits generally comes at a cost to somatic maintenance, such as immune performance (Hau 2007). We suggest, therefore, that our finding that plasma T was higher in birds with *ad libitum* food compared to birds that had experienced a period of food restriction is consistent with T‐mediated reproductive trade‐offs. Specifically, when energy is limited, T production and secretion may be suppressed to avoid the costs of T‐mediated male reproductive behaviours. In support of this suggestion, adult male Zebra Finches *Taeniopygia guttata* subjected to short‐term (4–10 h) food deprivation decreased plasma T, singing rate and courtship behaviour towards females (Lynn *et al*. 2010). Furthermore, spring snow storms, which likely reduced food availability and elevated thermoregulatory demands, were associated with reduced plasma T of free‐ranging male Song Sparrows *Melospiza melodia* (Wingfield 1985). If the hypothesis that T production and secretion are suppressed during periods of energy constraint to avoid the costs associated with T‐mediated male reproductive behaviours is correct, such a system would allow the reproductive morphology of birds to develop at approximately the correct time (as determined by photoperiod), but the characteristic male reproductive behaviours necessary for breeding may be inhibited due to low plasma T. Once food availability, and thus energetic status, is suitable, plasma T might increase, causing the expression of male reproductive behaviours and the commencement of breeding. Our finding that plasma T was still low after the reinstated *ad libitum* group had 2 weeks of *ad libitum* food access suggests, however, that there may be lasting (i.e. on the order of weeks) effects of poor energetic status on T production and secretion.

### Hypothalamic and Anterior Pituitary Gland Endocrine Activity

The mechanism(s) by which energetic status affects T production is/are unclear, but potentially involves modulation at multiple levels of the HPG axis. This mechanism may involve reduced gonadotropin production and/or secretion. We found that plasma LH did not change over the study in either the *ad libitum*‐fed birds or the birds subjected to 2 weeks of food restriction; by contrast, food restriction for 4 weeks caused plasma LH to decrease. This finding is in line with other food availability manipulation studies of wild and domesticated avian and mammalian species, which generally show that food restriction decreases plasma LH and FSH (Tanabe, Ogawa & Nakamura 1981; Hoshino *et al*. 1988; Lal *et al*. 1990; Hahn 1995; Kobayashi, Cockrem & Ishii 2002; Hill, Elmquist & Elias 2008; but see Perfito *et al*. 2008). Declines in plasma gonadotropins in response to negative energetic status appear to be due to decreases in the anterior pituitary gland content of their corresponding subunit mRNAs (Kobayashi, Cockrem & Ishii 2002; Kobayashi & Ishii 2002; Ciccone, Dunn & Sharp 2007), but it remains unclear whether energetic status directly modulates levels of these mRNA subunits. Indeed, food restriction also reduces the sensitivity of the pituitary gland gonadotrope to GnRH (Tanabe, Ogawa & Nakamura 1981).

Decreased production and secretion of LH may also result from impairment of the GnRH system. In laying hens, food restriction appears to reduce the releasable store of GnRH‐I in the ME (Bruggeman *et al*. 1998). Furthermore, providing laying hens with *ad libitum* food after a period of food restriction increased hypothalamic GnRH‐I mRNA (Ciccone, Dunn & Sharp 2007). In cockerels, food restriction decreased the *in vitro* baseline and depolarization‐induced release of GnRH‐I from isolated mediobasal hypothalami (Lal *et al*. 1990). Taken together, these studies in chickens suggest that food restriction inhibits production of GnRH‐I mRNA and release of the peptide in this species. However, we found no effect of food restriction on the hypothalamic GnRH system of Abert's Towhees or on the amount of their ME GnRH‐I‐ir. It is unclear whether results in domesticated species apply to free‐ranging birds. It is possible that changes in GnRH‐I expression similar to those observed in chickens occurred in food‐restricted towhees, but these changes are not reflected in hypothalamic levels of the peptide.

### The GnIH–NPY System

Alternatively, food availability may modulate the production and secretion of gonadotropins through effects on the GnIH–NPY system. There is evidence in seasonally breeding birds that GnIH controls reproductive activation through inhibitory actions on gonadotropes (Bentley *et al*. 2003; Calisi, Rizzo & Bentley 2008; Small *et al*. 2008b; Perfito *et al*. 2011). Furthermore, via their interactions with NPY cells in the IN, GnIH cells may modulate this inhibitory influence in response to energetic status (Clarke *et al*. 2009; Klingerman *et al*. 2011). In the present study, the area of GnIH‐ir perikarya was inversely related to the amount of time that birds experienced negative energetic status. In addition, food‐restricted birds had more IN NPY‐ir perikarya than *ad libitum*‐fed birds. These observations are consistent with the idea that negative energetic status was associated with upregulation of the NPY system that, in turn, promoted the release of GnIH. Enhanced GnIH secretion in food‐restricted birds may account for the reduced LH secretion and consequently plasma T in these birds.

Another neuropeptide whose activity is related to energetic status is GnRH‐II, a second form of GnRH that is highly conserved across almost all vertebrates (Schneider & Rissman 2008). In the Musk Shrew *Suncus murinus* midbrain levels of GnRH‐II show the opposite response to negative energetic status as compared to that of NPY: GnRH‐II content declined in response to food restriction and increased after refeeding (Kauffman *et al*. 2006). Crucially, these energetically aware GnRH‐II cells were associated with NPY‐ir fibres and, therefore, appear to work with NPY cells to coordinate the reproductive system response to energetic status (Bojkowska *et al*. 2008). Although, to our knowledge, experimental studies of the response of GnRH‐II to negative energetic status in birds are lacking, the available evidence in birds is consistent with a role similar to that in mammals (Maney, Richardson & Wingfield 1997; Stevenson & MacDougall‐Shackleton 2005; Stevenson *et al*. 2008; Perfito *et al*. 2011). In particular, GnRH‐II cells in the White‐crowned Sparrow *Zonotrichia leucophrys* possess putative GnIH receptors (Bentley *et al*. 2006). Thus, GnRH‐II may contribute to the reproductive system response to negative energetic status of birds. We suggest, therefore, that improving our understanding of the interactions between GnIH, NPY, GnRH‐II and their role in integrating information on energetic status into the reproductive system may shed light on the physiological mechanisms by which food availability influences the HPG axis.

### Gonadal GnIH

Finally, food availability may affect plasma T through gonadally produced GnIH. Avian testes express this peptide and its receptors, and experimental evidence supports an inhibitory role for GnIH in the control of T secretion (Bentley *et al*. 2009; McGuire & Bentley 2010; Tsutsui *et al*. 2012), including in response to signals of metabolic stress (McGuire, Koh & Bentley 2013). Overall, the effects of food availability on T production are potentially mediated at multiple levels of the HPG axis. Further research is needed, however, to shed light on the specific mechanism(s) involved.

### Secondary Sexual Characteristics

In contrast to the long‐term effects on T secretion, cloacal protuberance width appears to be highly sensitive to changes in energetic status. Indeed, 2 weeks of food restriction caused this organ to decrease in size. Furthermore, returning food‐restricted birds to *ad libitum* food availability for 2 weeks caused cloacal protuberance width to rapidly increase and reach a size similar to that of *ad libitum*‐fed birds. This increase, despite no associated change in plasma T, suggests that factors other than plasma T regulate development of the cloacal protuberance and that these factors are responsive to energetic status. The T metabolite DHT stimulates growth of the cloacal protuberance (Tramontin, Wingfield & Brenowitz 2003; Owen‐Ashley, Hasselquist & Wingfield 2004). We are aware of just one study that has examined plasma DHT in birds experiencing food restriction. In male Garden Warblers *Sylvia borin* caught during their spring migration, during which they also develop their gonads, food restriction reduced body mass but had no effect on plasma DHT (Bauchinger, Van't Hof & Biebach 2008). Therefore, further research is needed to examine whether DHT is also responsive to food availability.

## Conclusion

Many birds use food availability as a proximate environmental cue to optimally time seasonal reproductive development. However, despite appreciating the importance of this cue for decades, the physiological mechanisms by which information on food availability is integrated into the HPG axis remain unclear. We hypothesized that seasonal reproductive activation is constrained by energetic status. Indeed, we found that energetic status modulates activity of the HPG axis in male Abert's Towhees; however, the response is complex and appears to vary with the level of the HPG axis considered. Our results are consistent with a role for the GnIH–NPY system in integrating information on energetic status at the level of the hypothalamus. However, this does not appear to involve a modulation of the amount of hypothalamic GnRH‐I. There also appears to be a role for anterior pituitary gland and/or testicular endocrine function in modulating reproductive development in the light of energetic status. Despite no evidence that energetic status modulated testicular growth, plasma LH and T were reduced in response to poor energetic status. A further illustration of the complexity by which energetic status affects the reproductive system is that the cloacal protuberance – but not LH or T secretion – was responsive to returning food‐restricted birds to *ad libitum* food availability. Future research is warranted to elucidate the relative importance of the hypothalamus and the gonads in integrating information on energetic status.

## Supporting information


**Lay Summary**
