# Supplementary material for: Food availability, energetic constraints and reproductive development in a wild seasonally breeding songbird
Source: Funct Ecol. 2015 May 1;29(11):1421–34. doi: 10.1111/1365-2435.12448 (PMC4974902; doi:10.1111/1365-2435.12448)
Supplement: Supplementary file 1 — Lay Summary [file FEC-29-1421-s001.pdf]

Functional Ecology
British Ecological Society

Food availability and seasonal reproductive activation in a songbird

Scott Davies, Thomas Cros, Damien Richard, Simone L. Meddle, Kazuyoshi Tsutsui, and Pierre Deviche

Many animals have an outstanding ability to forecast when they need to be in breeding condition, yet much remains to be learned about how they achieve this. Most temperate zone songbirds, in particular, have to be exceptional forecasters because their reproductive system is shutdown during the winter and, to be able to breed, must be activated in the spring. Activation involves dramatic rises in the secretion of reproductive hormones and gonadal growth. This process takes time, so songbirds need to start it well before they need to breed and use cues from the environment that forecast when suitable environmental conditions are approaching. For decades, scientists have recognized that food availability is important because seasonal reproductive activation takes longer if birds are energetically constrained. However, our understanding of the physiological mechanisms that link food availability to activation of the reproductive system is limited.

To address this issue, we food-restricted captive adult male Abert's Towhees, *Melozone aberti,* during reproductive activation. We investigated whether energy deficiency constrains multiple aspects of reproductive activation, in particular the growth of the testes. Since testis growth is the culmination of an endocrine cascade that begins in the brain, we also aimed to understand which steps of this cascade are affected by energetic constraint and, to do so, measured endocrine activity at all three points of this cascade (i.e.,

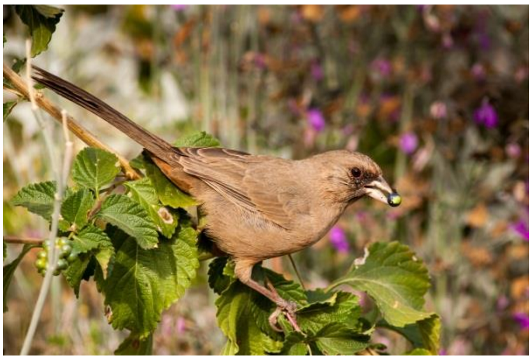

An Abert's Towhee. Photograph by Christofer Bang.

brain, anterior pituitary gland, and testes). Consistent with the majority of studies in songbirds, we found no effect of food restriction on testis growth. However, food restriction influenced plasma levels of hormones from all levels of the cascade responsible for reproductive activation.

These results suggest that energetic constraint modulates reproductive activation not through changes in the growth of the testes, but through changes in the plasma levels of hormones. In other words, even when constrained by energy deficiency, male songbirds can morphologically enter breeding condition, but delay increasing reproductive endocrine activity. This delay may be beneficial as it presumably decreases the expression of energetically costly behaviours, such as aggression and singing, which are stimulated by reproductive hormones.
